# The Impact of Milled Wood Waste Bottom Ash (WWBA) on the Properties of Conventional Concrete and Cement Hydration

**DOI:** 10.3390/ma16196498

**Published:** 2023-09-29

**Authors:** Marija Vaičienė, Jurgita Malaiškienė, Qaisar Maqbool

**Affiliations:** 1 Civil Engineering Faculty, Vilnius College of Technologies and Design, 10303 Vilnius, Lithuania; 2 Laboratory of Composite Materials, Faculty of Civil Engineering, Institute of Building Materials, Vilnius Gediminas Technical University, 08217 Vilnius, Lithuania; jurgita.malaiskiene@vilniustech.lt; 3 Department of Materials, Environmental Sciences and Urban Planning, Università Politecnica delle Marche—INSTM Research Unit, 60131 Ancona, Italy; q.maqbool@pm.univpm.it

**Keywords:** conventional concrete, milled wood waste bottom ash, physical and mechanical properties, concrete durability, freeze–thaw resistance, cement hydration

## Abstract

Wood waste bottom ash (WWBA) is a waste generated in power plants during the burning of forest residues to produce energy and heat. In 2019, approximately 19,800 tons of WWBA was generated only in Lithuania. WWBA is rarely recycled or reused and is mostly landfilled, which is both costly for the industry and unsustainable. This study presents a sustainable solution to replace a part of cement with WWBA at 3%, 6%, 9%, and 12% by weight. Problems are also associated with the use of this material, as WWBA could have a relatively large surface area and a high water demand. For the evaluation of the possibilities of WWBA use for cementitious materials, the calorimetry test for the cement paste as well as X-ray diffraction (XRD), thermography (TG, DTG), and porosity (MIP) for hardened cement paste with the results of physical and mechanical properties, and the freeze–thaw resistance of the concrete was measured and compared. It was found that WWBA with a large quantity of CO_2_ could be used as a microfiller with weak pozzolanic properties in the manufacture of cementitious materials. As a result, concrete containing 6% WWBA used to substitute cement has higher density, compressive strength at 28 days, and ultrasonic pulse velocity values. In terms of durability, it was verified that concrete modified with 3%, 6%, 9%, and 12% WWBA had a freeze–thaw resistance level of F150. The results show that the use of WWBA to replace cement is a valuable sustainable option for the production of conventional concrete and has a positive effect on durability.

## 1. Introduction

According to the United Nations Environment Programme (UNEP) report, in 2020 the construction and operation of buildings accounted for 36% of the global energy demand and 37% of global CO_2_ emissions. By far, the largest source of greenhouse gas emissions is related to the production of building materials and the operation of buildings [1]. European policies promote and stimulate green innovations in the reuse of waste as secondary raw materials to boost the market and new green business opportunities. Three materials: concrete, steel, and aluminium, are responsible for 23% of total global emissions [2].

Concrete is one of the most commonly used materials in the construction industry. Cement is the binding agent in concrete, the most widely used material on the earth [3]. Approximately 60% of the emissions from cement production are associated with the chemical calcination process, in which raw materials such as limestone are heated at high temperatures to release CO_2_ from the rock and produce clinker [4]. Cement production is considered responsible for approximately 7.4% of global CO_2_ emissions (2.9 Gtons in 2016) [5]. The authors report that 8.7 million tons of CO_2_ were found to be uptaken by mortars and concretes made with Portuguese cement over a ten-year period, in which 37.8 million tons were released due to the calcination process [6]. Therefore, steps are taken to reduce and phase out the production of CEM I cement and to expand the production of composite cements, in particular using secondary raw materials.

In recent times, sustainable methods have led to the exploration of alternative materials that can replace or supplement traditional cement. By substituting cement with supplementary materials such as fly ash, slag, and natural pozzolans, the carbon footprint is reduced, and improved mechanical properties and durability are often achieved [7]. As concrete endures as the scaffold of modern infrastructure, its evolution continues to intertwine with technological prowess, ecological consciousness, and a persistent quest for durability and performance.

Using waste as a partial replacement for cement and/or aggregates is a potential way to mitigate the negative environmental impact by reducing landfill space, carbon dioxide emissions, and the consumption of natural raw materials. Following the principles of circular economy, researchers attempt to minimise the negative impact by developing “green” concretes [8,9,10] containing reusable organic and inorganic industrial waste.

However, the introduction of sustainable technologies within construction and building materials has provoked an investigation of innovative materials that can enhance performance and alleviate environmental concerns [11]. One such material of burgeoning interest is milled wood waste bottom ash (WWBA), an often-disregarded residue arising from biomass combustion processes. WWBA has gained attention as a supplementary cementitious material due to its latent and pozzolanic hydraulic properties [12,13]. Pozzolanic materials, when finely divided and mixed with Ca(OH)_2_ (a by-product of cement hydration), react to form additional cementitious compounds, improving the durability and strength of the concrete. The hydraulic nature of WWBA suggests that it has the ability to react with Ca(OH)_2_ in the presence of water, forming hydration products similar to those of cement hydration. Furthermore, the latent hydraulicity of WWBA introduces complexities in the hydration kinetics, influencing the formation of the calcium silicate hydrate (CSH) gel, the primary binding phase in concrete. The resultant interactions within the microstructural fabric reshape the concrete’s pore structure and morphology, inevitably altering its long-term behaviour. This dual reactivity shows the potential of WWBA to be used as a potentially effective partial cement replacement [14,15].

However, it was found that the chemical composition of WWBA differed depending on the combusted materials and the incineration mode; however, the main oxides present in the WWBA are similar: (SiO_2_ + Al_2_O_3_ + Fe_2_O_3_ about 50%), CaO (about 20%) with loss on ignition (LOI) of about 20%, and small quantities of Na_2_O, MgO, K_2_O, etc. [16,17]. Other authors [18,19,20,21] report that WWBA is chemically composed of silica 4–40%, carbon 5–30%, calcium 7–33%, potassium 3–4%, magnesium 1–2%, manganese 0.3–1.3%, phosphorus 0.3–1.4%, sodium 0.2–0.5%, and up to 60% of organic matter. Due to its high carbon content, the use of WWBA is limited to low- and medium-strength concrete in the construction industry. However, it has great potential in masonry products, road paving materials, etc. [22]. The chemical and mineral composition of WWBA depends mainly on the incineration conditions. If the waste wood is incinerated at 700 °C and above, the carbonates are decomposed, the organic matter is completely burnt and the ash acquires pozzolanic properties [17,23]. Other researchers analysed the physical, chemical, and mechanical properties of biomass combustion ash to use it in concrete, mainly due to the pozzolanic properties of some types of bottom ash. Authors [24,25,26] reported that WWBA has pozzolanic properties, making the 28 and 365-day strength of concrete modified with up to 15% of bottom ash similar to the strength of control specimens (45 MPa) or approx. 10% higher due to lower interfacial porosity [27]. Other authors found that WWBA increases water demand, porosity, and therefore reduces the strength of concrete [14,28,29,30]. For example, replacing 20% of cement with WWBA caused the compressive strength at 28 days to reduce by approximately 10% [28]; at 28 days, the compressive strength of concrete was reduced by 50% (from approximately 42 MPa to 24 MPa); and at 90 days it decreased from 47 MPa to 37 MPa when 30% of cement was replaced with WWBA [29].

WWBA added at up to 10% does not have a negative effect on the workability of concrete; however, the slump flow of concrete reduces when the content of bottom ash is increased to 20% [25]. It was also found that 30% of the WWBA added increases the initial and final setting time of the concrete three times [31,32].

WWBA also increases the porosity of concrete [33] and reduces the resistance to alkali silica (ASR), especially when a relatively large amount of cement, namely 50% and 60%, was replaced with WWBA [32]. In particular, WWBA with high LOI absorbed more water and chemical admixtures resulting in increased slump loss, decreasing the air-entraining effect, and bleeding and decreasing the strength of concrete. Usually, the LOI values in WWBA are 0.5–25.0% by weight [34]. On the other hand, a decrease in pozzolanic reactions will take place as a result of the low fineness of the coarser carbon particles. A high LOI coupled with a low fineness and quality of WWBA would negatively affect the development of concrete strength [35].

It should be noted that the possibilities of reusing wood bottom ash have not been studied as extensively as other types of ash used as supplementary binding materials. However, there are studies on the reuse of wood bottom ash in the production of self-compacting concrete [36,37,38], stoneware tiles [39], aggregates [40,41], and soil stabilization [42].

The aim of this work is to find ways of reducing cement content in normal fiber concrete mixes by substituting it with WWBA (consisting high amount of LOI, about 53.7% of CO_2_) and determine effect of WWBA on cement hydration, physical and mechanical properties, and durability of concrete.

## 2. Materials and Methods

### 2.1. Raw Materials and Their Properties

**Figure 1:**
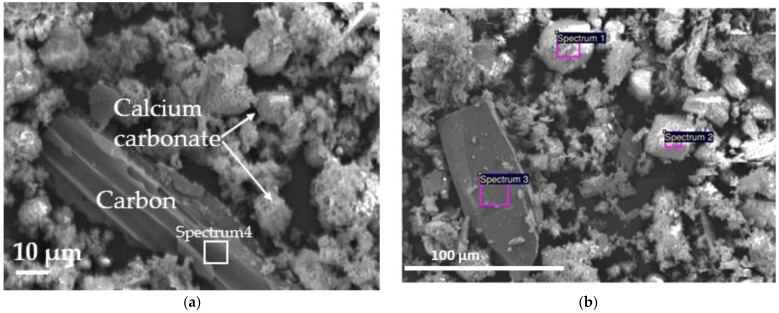
Images of WWBA: (**a**) SEM image, (**b**) SEM image for EDS analysis.

Cement CEM I 42.5, wood waste bottom ash (WWBA), natural sand, and crushed granite were used for the preparation of concrete specimens. The cement paste samples for the analysis of cement hydration with WWBA were prepared of cement, WWBA (0%, 6% and 12% and marked B0, B6, B12), and water (W/B 0.35). The mineral composition of the cement was as follows: 56.6% of C_3_S, 16.7% of C_2_S, 9.0% of C_3_A, 10.6% of C_4_AF, and 7.1% of other substances (alkaline sulphates and CaO). The d_50_ of cement particles was 10.3 µm and the d_90_—22.9 µm. The average diameter of cement particles was 11.7 µm, obtained by laser diffraction (CILAS 1090, range 0.10–500.0 µm). The cement particle density was 3.1 g/cm^3^; the bulk density was 1.1 g/cm^3^. The chemical compositions of cement and WWBA powders determined by X-ray fluorescence (XRF, Rigaku ZSX Primus IV) are presented in Table 1. The scanning electron microscope (SEM) images and X-ray microanalysis performed by the energy dispersion spectrometer (EDS) of WWBA are presented in Figure 1 and Table 2. SEM analysis was performed using a JSM-7600F scanning electron microscope (JEOL, Tokyo, Japan). The analysis was performed at an accelerating voltage of 10 kV and the secondary electron mode was used in image formation. Before the investigation, the surface of WWBA powders was covered with a layer of electrically conducting material using a QUORUM Q150R ES device (Quorum Technologies Ltd., Darmstadt, Germany). X-ray microanalysis was performed by Inca Energy 350 energy dispersion spectrometer (Oxford Instruments, Abingdon, UK), using the Silicon Drift type detector X-Max20 (Oxford Instruments, Abingdon, UK). The INCA Software package (INCA Suite 4.04, Oxford Instruments) was used.

WWBA, according to chemical composition, contains 22.7% of CaO, 53.7% of CO_2_, and other substances. A relatively high amount of CO_2_ in WWBA indicates the presence of carbonates, mainly CaCO_3_, which is active and accelerates cement hydration [43,44]. WWBA also contains residual organic matter that most often retards cement hydration [45,46].

The elemental analysis shows that in WWBA some particles of calcium carbonate (Spectrum 2), carbon (Spectrum 4), sand (Spectrum 3), and compounds from various elements (Spectrum 1) were identified. This made it interesting to research how WWBA of this composition modifies the properties of conventional concrete and cement hydration. More than 10% organic matter in the ash indicates that the incineration process is not optimal and needs to be changed or optimised [47]. The WWBA used was obtained by burning waste wood at 1000–1200 °C for approximately 30 min, but this time, when large quantities of biomass are burnt, it was not sufficient for the complete combustion of raw materials. SEM images shows (Figure 1), that WWBA consists of various shape materials: cubic (usually calcium carbonate), irregular form plates (sand and carbon), sticks, balls, and very fine powders, which covered coarser compounds.

**Figure 2:**
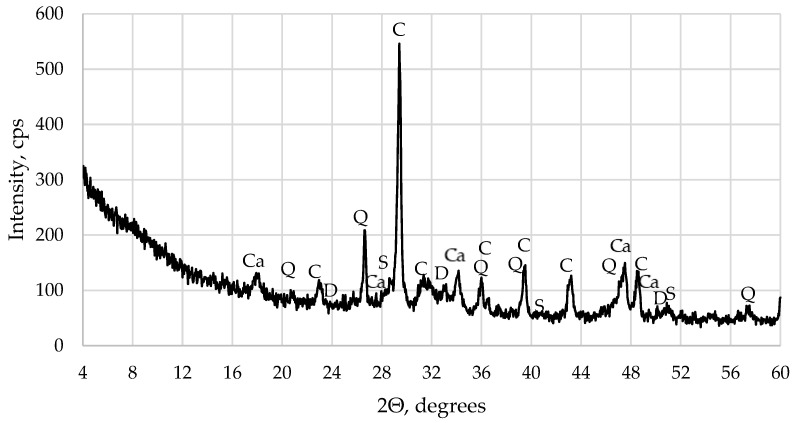
WWBA X-ray diffraction pattern. (Ca—Ca(OH)_2_ prevails; Q—quartz; C—calcite; S—sylvite; D—dolomite).

XRD analysis of WWBA powders (Figure 2) confirmed the prevalence of calcite in the ash, with small amounts of quartz, dolomite, portlandite, and sylvite also identified. The low amount of portlandite could be identified because WWBA were tested after 3 months of keeping them in natural conditions so hydration of CaO could occur. On contact with water or moisture, up to 50% of the CaO content in WWBA will undergo a chemical reaction to form Ca(OH)_2_, which will subsequently result in the development of CaCO_3_ when it comes into contact with carbon dioxide (CO_2_) [35].

The activity of WWBA determined by the Chapelle method [48] (standard NF P18-513 [49]) is 410 mg/g. The activity value was low because this method reveals the content of early SiO_2_ better, whereas in our tests CaO and CO_2_ prevail in WWBA. The physical properties of the WWBA are given in Table 3. The average diameter was obtained by laser diffraction (CILAS 1090, range 0.10–500.0 µm).

The physical properties of the sand are given in Table 4. Crushed granite of two fractions was used as a coarse aggregate: fraction 5/8, bulk density 1.3 g/cm^3^ and fraction 11/16, bulk density 1.4 g/cm^3^.

A superplasticizer (SP) based on polycarboxylic ether polymers (Glenium 560) was used in all mixtures. SP density was 1.0 g/cm^3^, and the pH value of 20% solution at 20 °C temperature was 5.6. All specimens were prepared with tap water according to the requirements of European standard EN 1008 [50].

The physical and mechanical properties of the polypropylene fibre (PP) Crackstop F6 Adfil NV are given in Table 5. Polypropylene fibrem measured according to EN 14889-2 [51], was used to prepare the concrete.

### 2.2. Mix Design and Specimen Preparation

Five concrete mixtures with designation codes BP0, BP3, BP6, BP9, and BP12 (BP designation means—bottom ash and polymer fiber concrete with different amounts of biomass bottom ash) were mixed. Concrete mixtures were formulated by partially replacing cement with WWBA in the range of 0% to 12%, by weight, step 3%. The BP0 samples were used as control samples. Table 6 presents the concrete mixture compositions and the slump flow of each mixture. It was determined that up to 12% of WWBA did not have a significant influence on the flow of the concrete mixture.

Cube-shaped specimens (100 × 100 × 100 mm) formed from the prepared mixtures were kept under normal conditions in the moulds for 1 day and for 27 days immersed in water at 20 ± 2 °C. For the concrete physical, mechanical, and durability tests we used 3 samples. The concrete specimens were made and cured for strength tests according to EN 12390-2 [52]. The compressive strength of concrete specimens was determined according to EN 12390-3 [53], and the density was determined according to EN 12390-7 [54] (3 specimens for each composition). To determine the water absorption rate, the specimens were dried at 60 °C to a constant weight (constant mass was reached when after 2 subsequent weightings, the final weight does not change more than 0.1%), then immersed into water of 20 °C and weighted after 10, 30, 60 min, 24 h, and 48 h.

Ultrasonic pulse velocity (UPV) was measured with a Pundit 7 device with two 54 kHz transducers. The ultrasonic pulse velocity was calculated from Equation (1):

(1) $$UPV=\frac{l}{\tau }$$

where: *l* is the length of ultrasonic pulse path through the specimen, i.e., the distance between the two transducers, which in this case was the length of the specimen; m (0.10 m for concrete specimens) and *τ* is the time of travel recorded by the test equipment, s.

The durability of concrete specimens (100 × 100 × 100 mm) was determined at 28 days of curing according to the requirements of LST 1428-17 [55]. The rapid freeze–thaw resistance method was used by freezing the water-saturated concrete specimens in air and thawing them in water. For rapid freezing and thawing, the specimens were soaked in 3% NaCl solution. This method was also chosen to determine the concrete resistance on NaCl. Before testing, saturated samples were removed from the water bath and placed in the manner that provides draining of the water. After 2 to 4 h, the reference specimens were tested to determine the initial compressive strength according to LST EN 12390-3 [53]. The remaining concrete specimens were placed in the freezing chamber (Rumed 3301). The freezing of the specimens in the air would last at least two hours. The temperature in the freezing chamber would be approximately (−18 ± 2) °C. After the freeze–thaw resistance test, the specimens were removed from the freezing chamber and placed in the bath with the salt solution of (18 ± 5) °C. The time to keep the specimens in the salt solution bath would not be less than (2 ± 0.5) h. If, after the required number of freeze–thaw cycles, the drop in compressive strength did not exceed 5%, the concrete was deemed to have passed the freeze–thaw test. After 150 cycles, the specimens were crushed, and the reduction in strength was calculated. According to the LST 1974 standard [56], if the compressive strength of the samples is appropriate, they are of the F150 class and could be used for horizontal concrete surfaces exposed to cold and rain.

To understand the influence of WWBA on cement hydration, samples (40 × 40 × 40 mm) of three additional mixtures B0, B6, and B12 were prepared without aggregates. The mineral composition and microstructure of the products present in the hardened pastes were studied by X-ray diffraction (XRD) and thermogravimetric analysis (TGA) after 7 and 28 days of curing in water. Hydration was stopped by immersing crushed samples in acetone for 72 h and then drying them in an electric oven at 50 °C for 24 h. Anatase was used as the internal standard for XRD analysis of concrete specimens with the binder: anatase ratio of 9:1. The height of the peaks of all diffraction patterns was adjusted in order to have the same intensity of the main anatase peak (2θ = 25.28°) in all diffraction patterns so that the relative intensity of the other peaks would be comparable. Thermal analysis (TGA) was performed with a PerkinElmer TGA 4000 thermal analyser (Rodgau, Germany). Samples with a weight of 50–60 mg were placed in a platinum crucible and heated at 10 °C/min in a nitrogen environment up to 1000 °C. The amount of portlandite was calculated according to the author [57] the mass loss within the 430–560 °C temperature range.

The amount of heat released during the hydration of the binder and the heat release rate were measured with the TONICAL III calorimeter (Toni Technik GmbH, Berlin, Germany). The mixtures (35 g of water and 100 g of solid substance) were analysed for 48 h at the operating temperature of 20 °C, and the heat evolution curves were recorded.

The Mercury intrusion porosimetry (MIP) method was applied to analyse the parameters of the cement paste pore structure using the Quantachrome Poremaster 33/60 pore analyser (Quantachrome Instruments, Boynton Beach, FL, USA) with a maximum pressure of 33,000 psi for a diameter of the pores ranging from 1100 μm to 0.0035 μm and with two low-pressure stations plus one high-pressure station. The specimens were carefully peeled from (40 × 40 × 40 mm) the samples middle.

## 3. Results

### 3.1. Analysis of Physical, Mechanical Properties and Durability of Concrete

**Figure 3:**
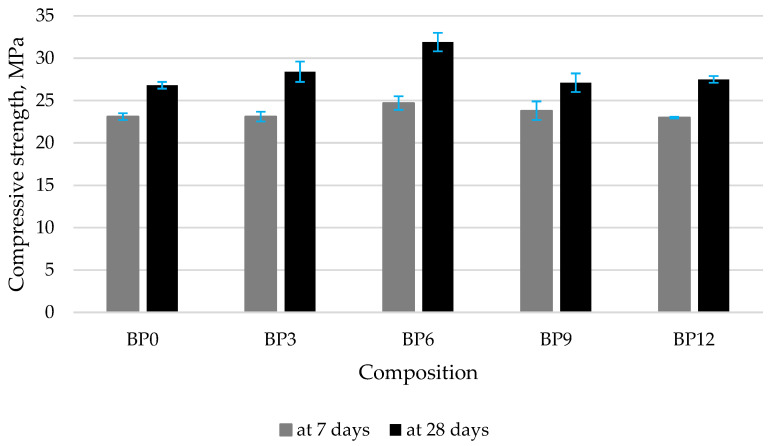
Compressive strength of concrete.

Concrete specimens containing 6% of WWBA had the highest compressive strength (Figure 3). At 7 days, similar compressive strength values were obtained in all batches; however, at 28 days, a 16% increase in compressive strength was observed in the specimens modified with 6% of WWBA. The compressive strength started decreasing with the further increase in WWBA content but remained similar to the strength of control specimens. According to the author [58], the compressive strength increased by 14.8% when 10% of the cement was replaced with wood ash. However, when 20% of the cement was replaced, the compressive strength dropped by 10.1%. Other authors [59] found that the compressive strength difference between control specimens and the specimens containing different amounts of alternative binders tended to decrease with a longer curing time. At 28 days of curing, concretes with 5%, 10%, and 15% cement replacement levels had compressive strengths equal to 93%, 78%, and 68% of the control specimen’s compressive strength, respectively. Similar results were reported by other researchers [60], who obtained the highest strength values of 24.7 MPa in the specimens where 10% of cement was replaced with wood ash. When part of the cement was replaced with WWBA, the compressive strength could increase due to several reasons: (1) high pozzolanic activity of WWBA (high calcite content and potentially active SiO_2_) [61] or the micro filling capacity of WWBA (average particle size is 24.2 µm).

A certain amount of WWBA used, because of low average particle size, may reduce the porosity of the specimens; however, a higher amount of WWBA increases water demand, and thus the porosity may increase.

**Figure 4:**
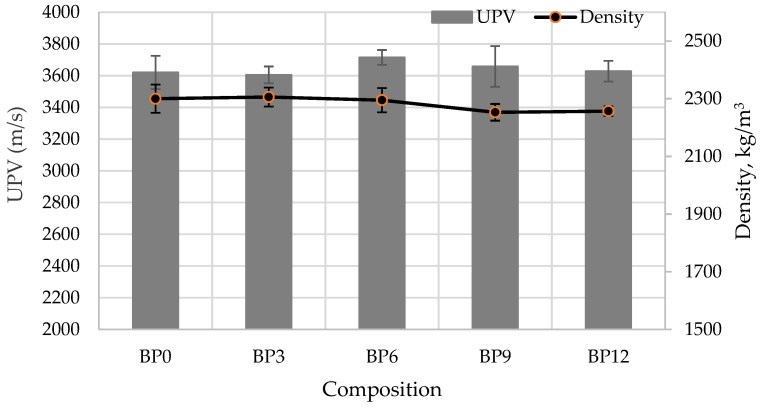
UPV and density of concrete.

The samples of composition BP6 demonstrated some of the highest density (approx. 2300 kg/m^3^) and ultrasound pulse velocity UPV (3715 m/s) values (Figure 4). Similar results were reported by authors [62]; the replacement of cement with 5% bottom ash produced the density values 2313 kg/m^3^. Compared to the control sample, BP6 had the same density and 3% higher UPV values. This means that concrete specimens where 6% of the cement is replaced with WWBA retain the same dense structure. With the addition of 9% WWBA, the density and UPV values started to reduce, and more pores and capillaries were formed. Such changes in concrete structure can be explained by the increased water demand by WWBA, the lower density of WWBA compared to cement density, and the possible change in the rate and progress of cement hydration. Similar results were obtained by the authors [63]. They established that the highest density, ultrasound pulse velocity, and compressive strength of concrete are the highest, when 5% of cement were replaced by biomass combustion fly ash (BM-FA). The explanation of such results was that in this composition, concrete contained much less free water, which affects the development of open pores. In the work [64] it was determined that replacement of up to 10% of cement with BM-FA the paste exhibits better rheological properties, not only lower yield stress but also lower viscosity and helped retain plasticizing effect. Then, the concrete has a higher density and a lower open porosity. Additionally, it has often been reported [35], that the presence of dominant fine particles in cementitious materials will lead to more water demand due to their larger surface area.

**Figure 5:**
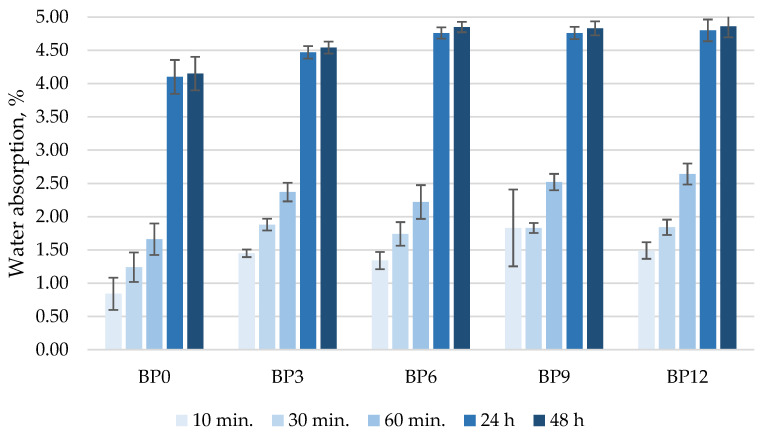
Water absorption kinetics.

Figure 5 illustrates the water absorption results of concrete samples cured in water for 28 days. It should be noted that the water absorption rate reached 1.8% during the first 10 min, 1.9% after 30 min, 2.6% after 60 min, and after 24 h, the specimens absorbed approximately 4.8% of water by the weight of the specimen. Water absorption followed the same trend, and after 48 h it was 4.9%. The water absorption kinetics test revealed that the samples without WWBA had lower water absorption values compared to other samples and that this could be due to the porosity of the WWBA particles, the high surface area, and the ability to absorb water.

According to the evaluation of freeze–thaw resistance (Table 7), all concrete specimens passed the resistance test of 150 cycles. Analysis of the results of compressive strength tests after freeze–thaw cycles revealed that the compressive strength of all compositions increased due to subsequent cement hydration, which can occur during thawing of the specimens in water at 20 °C. The most significant increase of 8.8% in compressive strength was observed in samples containing 6% WWBA, due to this concrete series, lower higher density, UPV, and compressive strength. No defects (cracking, scaling, etc.) were observed in the samples after freeze–thaw test cycles.

In summary, the freeze–thaw resistance test results show that concrete specimens with varying amounts of WWBA can be used in structures with freeze–thaw resistance requirements up to grade F150.

### 3.2. The Impact of WWBA on Cement Hydration

**Figure 6:**
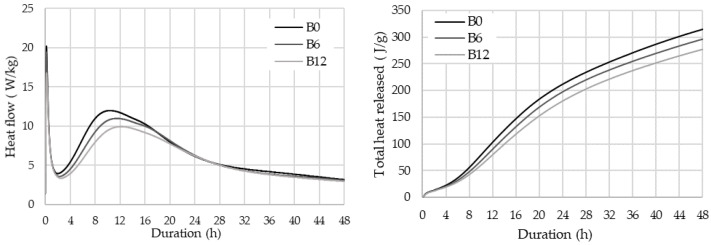
Impact of WWBA on heat flow and total heat released by cement.

The calorimetry tests (Figure 6, Table 8 and Table 9) showed that WWBA retarded cement hydration: the peak of the third stage of hydration was reached 75 min (B6) and 108 min (B12) slower, while after 48 h the heat of hydration and the degree of hydration in the samples modified with WWBA reduced 6% and 12%, respectively, depending on the reduced cement content in the mix. These results show that during the first 48 h WWBA has no effect on cement hydration and acts as an inert additive; the amount of released heat drops by the same percentage as the percent of replaced cement.

**Figure 7:**
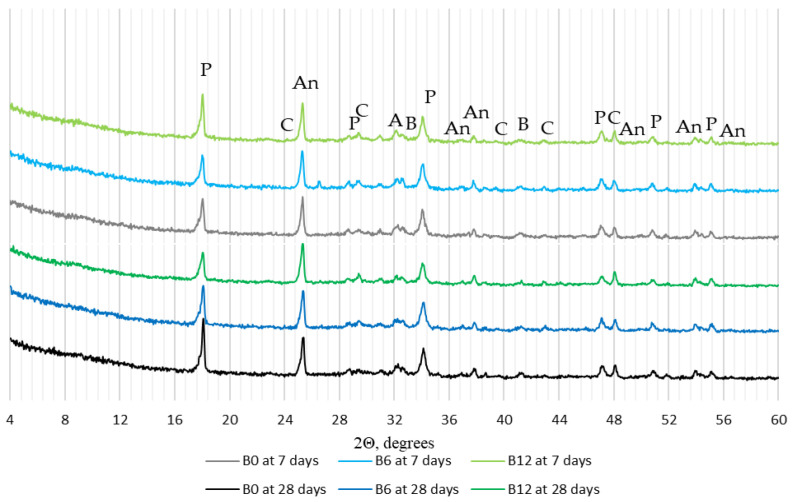
X-ray diffraction patterns of hardened cement paste at 7 and 28 days (P—portlandite, C—calcite, An—anatase, A—alite, B—belite).

According to X-ray analysis (Figure 7), the amount of portlandite decreased in WWBA modified specimens modified with WWBA (peak intensity decreased at approximately 18° and 34°), and this trend was expressed more after 28 days of curing. The test results also revealed a lower intensity of the main peak of alite (approx. at 32°). These results suggest that WWBA has binding properties and acts as a pozzolanic additive; on the other hand, the intensities of these minerals could have been reduced due to the lower cement content in the mixture. Therefore, the thermal analysis of selected specimens B0, B6, and B12 was carried out to determine the amount of portlandite.

**Figure 8:**
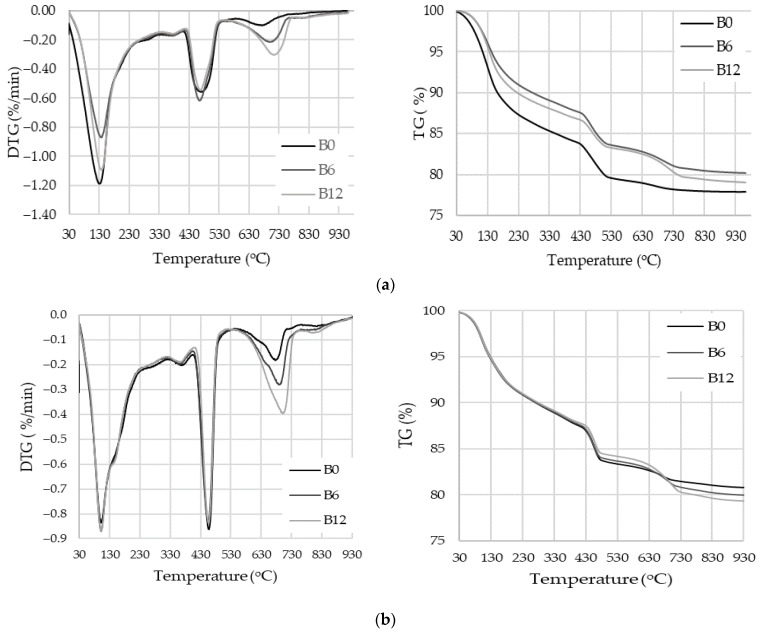
TG and DTG of hardened cement paste: (**a**) at 7 days; (**b**) at 28 days.

The results of the thermal analysis revealed low WWBA activity after 7 days of curing because the portlandite content (Figure 8a), calculated by the same amount of cement, was slightly lower, namely 21.9% in B6 and 20.3% in B12, compared to 22.6% in the control specimens B0 (Table 10). According to the results after 28 days of curing (Figure 8b, Table 10), the portlandite content in all compositions was very similar to that in the control specimen (18.7%) or even increased to 19.4% in compositions with the highest WWBA content (12%). Compared to control specimens, WWBA specimens showed slightly lower mass loss at 110–350 °C. This means that bottom ash does not accelerate CSH and CASH formation in the first 7 days, compared to the control sample. At 28 days, the specimens of all compositions had a similar mass loss in the 110–170 °C temperature range when CSH decomposition occured. These results suggest that WWBA may have a weak pozzolanic effect, because in B6 and B12 the cement content is less. Similar results were shown in the [12] article, in which it is concluded that the reduction in portlandite with increasing replacement of cement with biomass ash is mainly due to its consumption by the pozzolanic reaction due to the dilution of cement to form hydration products such as CSH.

**Figure 9:**
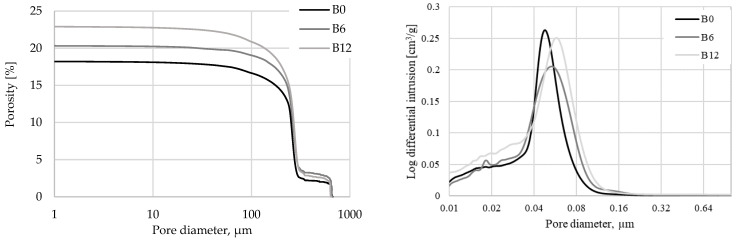
Porosity results.

The Mercury Intrusion Porosimetry (MIP) test showed that WWBA increases the porosity of hardened cement paste (Figure 9): with the addition of WWBA at 6%, the porosity of hardened cement paste increased 11.5% and with 12% of WWBA the porosity increases 25.8%. WWBA also caused the average diameter of the pores to increase from 0.046 µm (B0) to 0.059 µm (B6) and to 0.049 µm (B12). Although the diameter of most of the pores in samples B6 increased, these pores were lower in quantity compared to the control specimen, while the number of pores with diameter below 0.04 µm was significantly lower. This result explains why samples B6 had the highest density and UPV values, especially compared to B0 and B12. In the literature [65], it is written that the use of WWBA as a filler in cement-based materials is possible when the size limits are such as 250 µm. Performance in cement paste is influenced by two known mechanisms, namely particle packing and filler effect phenomena. Additionally, the added WWBA particles would act as a nucleation zone after their integration into the cement paste, resulting in improved hydration and subsequently the enhancement of compressive strength [35]. The increase in the general porosity of WWBA-produced cementitious materials could be caused by irregular shaped particles of ash that contain numerous pores not filled by the hydration products after curing and subsequent hardening, but micropores in the size range of 0.01–0.1 µm could greatly increase due to the wide distribution of particles and irregular particle structures which seem to fill the pores [66].

Rajamma [29] studied porosity and pore diameter using mercury intrusion porosimetry (MIP). The results also showed an increase in total porosity with increasing wood ash replacement, although the median pore diameters decreased. It was explained that the mean diameter decreased as a result of the finer particle inclusion, which acted as a better packing filling effect. However, porosity increased due to less hydration in mortars with wood ash [23].

## 4. Conclusions

WWBA with an average particle size of about 60 µm and a large quantity of CO_2_ could be used as a microfiller with weak pozzolanic properties in the manufacture of cementitious materials. This additive work as inert and has no effect on cement hydration in the first 48 h; however, at 7 days, WWBA, which contains approximately 50% CO_2_, may have a weak pozzolanic activity as a result of reduced quantities of portlandite and alite, as well as a mass loss very similar to that of the control sample in the temperature range when the decomposition of CSH and CASH occurs.

The highest compressive strength values were obtained in the samples in which 6% of the cement was replaced with WWBA. These specimens also had the highest density and UPV values in comparison to other compositions as a result of particle packing, filler effect phenomena, and improved hydration. The MIP results showed that the hardened cement paste samples of this composition had a higher total porosity than the control samples due to the irregular shape of the WWBA particles, but they also had a higher quantity of pores smaller than 0.04 µm, because of capability to improve cement hydration.

The freeze–thaw resistance tests showed that concrete samples modified with 3%, 6%, 9%, and 12% WWBA maintained compressive strength characteristics after 150 freeze–thaw cycles and therefore can be used in structures where freeze–thaw resistance of F150 is required for structural concrete. The highest increase in compressive strength (8.8%) after freeze–thaw cycles due to the highest ultrasound pulse velocity, density, and strength before freezing was obtained for concrete samples in which 6% of the cement was replaced by WWBA.

## Figures and Tables

**Table 1:** Chemical composition of CEM I 42.5 and WWBA (wt%).

Compound	Chemical Composition (wt%)	
	CEM I 42.5 R	WWBA
SiO_2_	20.4	8.34
Al_2_O_3_	4.0	1.54
Fe_2_O_3_	3.6	0.95
CaO	63.2	22.7
MgO	2.4	2.57
SO_3_	3.1	2.79
K_2_O	0.9	3.78
Na_2_O	0.2	0.21
Cl	0.05	0.27
CO_2_	-	53.7

**Table 2:** Elemental composition of WWBA (wt%) Spectrum 1–Spectrum 3 (Figure 1b).

Element	Elemental Composition (wt%)			
	Spectrum 1	Spectrum 2	Spectrum 3	Spectrum 4
C	12.9	25.0	9.60	91.6
O	50.5	48.1	54.0	4.0
Na	0.60	0.12	–	
Mg	1.37	0.58	–	
Al	3.03	0.06	–	
Si	10.0	0.19	35.3	
P	1.25	0.13	–	
K	3.31	1.58	–	2.97
Ca	14.2	22.1	1.20	
Mn	2.82	2.22	–	
Cl	–	–	–	1.42

**Table 3:** Properties of the WWBA.

Bulk Density, g/cm^3^	Water Absorption, %	d_50_, µm	d_90_, µm	Average Diameter, µm
0.6	44.1	16.2	59.7	24.2

**Table 4:** Properties of the fine aggregate.

Fraction	Characteristics		
	Particle Density, g/cm^3^	Bulk Density, g/cm^3^	Water Absorption, %
0/2	2.4	1.6	0.6

**Table 5:** Properties of the polypropylene fibre (PP).

Density, kg/dm^3^	Equivalent Diameter, mm	Length, mm	Tensile Strength, MPa
0.91	0.78	6	110

**Table 6:** Compositions of concrete mixes (kg/m^3^) and flow of the mixtures.

Mix Designation	Binders (Cement + WWBA)		Crushed Granite	Sand	PP Fiber	SP	W/B	Flow, mm
	Cement	WWBA						
BP0	300	0	1000	980	0.9	3.0	0.55	420
BP3	291	9	1000	980	0.9	3.0	0.55	420
BP6	282	18	1000	980	0.9	3.0	0.55	420
BP9	273	27	1000	980	0.9	3.0	0.55	420
BP12	264	36	1000	980	0.9	3.0	0.55	410

Note: 50% of 5/8 fraction and 50% of 11/16 fraction crushed granite.

**Table 7:** Freeze–thaw resistance results.

Designation of Concrete	BP0	BP3	BP6	BP9	BP12
Change in compressive strength, %	+5.5	+7.0	+8.8	+4.5	+3.3
Appearance of specimens	No visible defects				
Number of cycles	150				

**Table 8:** Time and heat of hydration of the tested pastes.

Paste Designation	Time of the Second Maximum (h)	Heat after Hours of Hydration (J/g)			
		12	24	36	48
B0	10 h 00 min	103.3	211.9	270.5	314.6
B6	11 h 15 min	90.3	197.5	254.6	296.2
B12	11 h 48 min	80.1	180.5	234.7	277.1

**Table 9:** Degree of hydration of the tested pastes.

Paste Designation	Degree of Hydration (-)			
	12	24	36	48
B0	32.8	67.4	86.0	100.0
B6	28.7	62.8	80.9	94.2
B12	25.5	57.4	74.6	88.1

**Table 10:** Mass loss in decomposition temperature ranges and the calculated quantity of portlandite.

Designation	Change in Weight, 110–170 °C, %	Change in Weight, 180–350 °C, %	Change in Weight, 430–560 °C, %	Portlandite Content in a Dry Specimen, %	Portlandite Content at the Equal Quantity of Cement, %	Change in Weight, 690–850 °C, %
After 7 days						
B0	5.91	4.08	4.36	22.6	22.6	0.49
B6	4.50	3.96	4.16	20.6	21.9	1.45
B12	5.50	3.84	3.60	17.9	20.3	2.12
After 28 days						
B0	3.49	3.73	3.77	18.7	18.7	0.80
B6	3.47	3.53	3.60	17.8	18.9	1.35
B12	3.51	3.52	3.47	17.1	19.4	2.03

## Data Availability

Not applicable.
